# Intraoperative transoesophageal echocardiographic detection of guidewire malposition and incomplete valve expansion in valve-in-valve transcatheter aortic valve implantation for Trifecta bioprosthesis failure: a case report

**DOI:** 10.1093/ehjcr/ytag183

**Published:** 2026-03-10

**Authors:** Ken-ichi Watanabe, Nagataka Yoshihara, Hirokuni Akahori, Taichi Sakaguchi

**Affiliations:** Department of Cardiovascular Surgery, Hyogo Medical University, 1-1 Mukogawa-cho, Nishinomiya City, Hyogo 663-8501, Japan; Department of Cardiovascular and Renal Medicine, Hyogo Medical University, 1-1 Mukogawa-cho, Nishinomiya City, Hyogo 663-8501, Japan; Department of Cardiovascular and Renal Medicine, Hyogo Medical University, 1-1 Mukogawa-cho, Nishinomiya City, Hyogo 663-8501, Japan; Department of Cardiovascular Surgery, Hyogo Medical University, 1-1 Mukogawa-cho, Nishinomiya City, Hyogo 663-8501, Japan

**Keywords:** Valve-in-valve TAVI, Transoesophageal echocardiography, Trifecta bioprosthesis, Guidewire malposition, Case report

## Abstract

**Background:**

Valve-in-valve transcatheter aortic valve implantation (ViV-TAVI) is widely used for failed surgical bioprostheses; however, procedural pitfalls remain, particularly in valves prone to early structural deterioration.

**Case summary:**

An 84-year-old woman with a failed 21 mm Trifecta bioprosthesis underwent ViV-TAVI. Although the guidewire crossed smoothly and fluoroscopy appeared reassuring, incomplete valve expansion occurred. Intraoperative transoesophageal echocardiography revealed that the guidewire had passed between degenerated leaflets rather than through the true central orifice. After repositioning under transoesophageal echocardiography guidance, full symmetric valve expansion and optimal haemodynamics were achieved.

**Discussion:**

This case highlights the risk of false procedural reassurance during ViV-TAVI and underscores the importance of actively confirming central guidewire trajectory, particularly in failed Trifecta bioprostheses.

Learning points

- Smooth guidewire passage does not exclude malposition in valve-in-valve transcatheter aortic valve implantation.
- Failed Trifecta bioprostheses are prone to leaflet tears, leading to false reassurance.
- Intraoperative transoesophageal echocardiography should actively confirm central wire trajectory before deployment.

## Introduction

Valve-in-valve (ViV) transcatheter aortic valve implantation (TAVI) is an established treatment option for patients with failed surgical bioprostheses who are at high or prohibitive surgical risk.^1^ Despite advances in device technology and imaging, procedural pitfalls remain, particularly in valves prone to early structural valve deterioration, such as the Trifecta bioprosthesis.

## Summary figure

**Figure:**
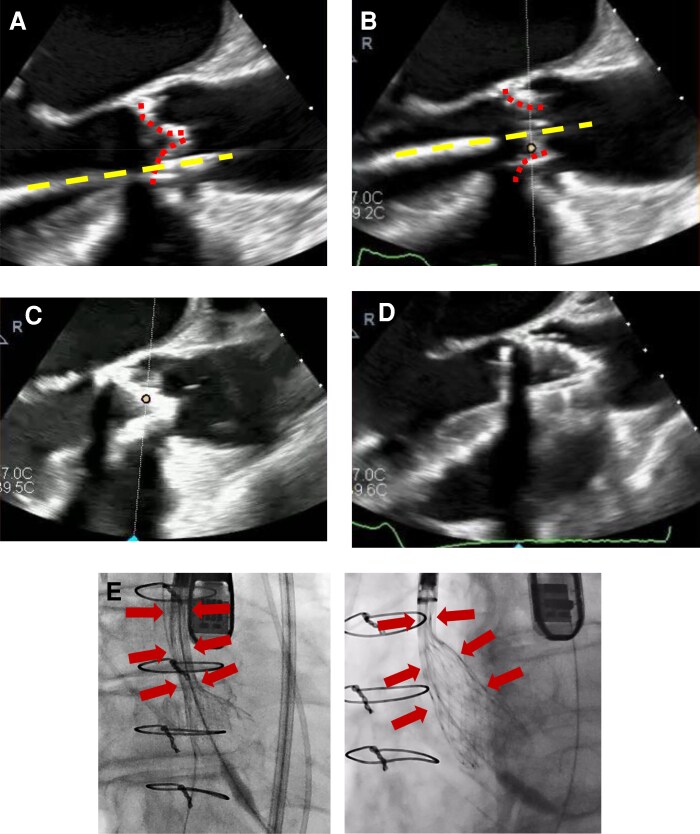
Intraoperative identification of guidewire malposition and its impact on valve expansion during valve-in-valve TAVI. (*A*) Mid-oesophageal long-axis transoesophageal echocardiography (TEE) showing the guidewire coursing between degenerated bioprosthetic leaflets, rather than through the true central orifice (dashed yellow line). (*B*) TEE image during initial deployment showing incomplete and asymmetric expansion of the transcatheter valve.(*C*) After repositioning, TEE demonstrates the guidewire passing through the true valve orifice, aligned with the prosthetic valve plane. (*D*) TEE image after guidewire repositioning showing full and symmetric valve expansion. (*E*) Fluoroscopic image demonstrating under-expanded valve configuration despite apparently acceptable wire position. (*F*) Fluoroscopic image after correction, confirming complete valve expansion.

## Case presentation

An 84-year-old woman with New York Heart Association Class III dyspnoea presented 10 years after surgical aortic valve replacement with a 21 mm Trifecta bioprosthesis. Transthoracic echocardiography demonstrated severe transvalvular regurgitation with moderate stenosis. Computed tomography showed leaflet calcification and an internal diameter of 22.2 mm but no definitive leaflet tear. Given her prohibitive surgical risk (STS score 31%), ViV-TAVI was planned.

During transfemoral access, the guidewire advanced across the prosthetic valve smoothly without resistance, and deployment of a self-expanding transcatheter valve was attempted. However, the valve failed to expand adequately and was recaptured. Focused review of intraoperative transoesophageal echocardiographic (TEE) revealed that the guidewire had passed between degenerated bioprosthetic leaflets rather than through the true central orifice. After repositioning the guidewire under real-time TEE guidance, redeployment achieved full and symmetric valve expansion. Post-procedural TEE confirmed optimal haemodynamics with no residual regurgitation. The postoperative course was uneventful, and the patient remained asymptomatic at 6-month follow-up.

## Discussion

This case does not propose a novel TEE technique. Rather, it highlights a critical procedural pitfall during ViV TAVI: guidewire malposition may occur despite smooth wire passage, reassuring fluoroscopic appearance, and non-diagnostic preprocedural computed tomography, particularly in failed Trifecta bioprostheses. The primary value of this report therefore lies in its preventive and cautionary message, emphasizing the risk of false procedural reassurance.

The Trifecta bioprosthesis is prone to early structural valve deterioration, frequently due to leaflet tears near the commissural posts.^2^ Such leaflet injury may be subtle or occult on preprocedural imaging yet can alter intravalvular anatomy, allowing the guidewire to track between leaflets rather than through the true central orifice. From a practical standpoint, mid-oesophageal long- and short-axis TEE views were particularly helpful in identifying wire trajectory relative to leaflet planes. Systematic interrogation of wire position before deployment and a low threshold for reassessment when valve expansion is incomplete are essential.

This report does not advocate a new TEE protocol but demonstrates a specific application of standard intraoperative TEE that may be underutilized during ViV-TAVI. A simple checklist approach may enhance safety in degenerated surgical bioprostheses.

## Limitations

This report is limited by its single-case nature and short follow-up duration. Consequently, the frequency of this complication and predictive markers cannot be determined.

## Data Availability

The data underlying this article are available from the corresponding author upon reasonable request.

## Lead author biography

Ken-ichi Watanabe, MD, PhD, is a cardiovascular surgeon and lecturer in the Department of Cardiovascular Surgery at Hyogo Medical University, Japan. He graduated from Hirosaki University School of Medicine and earned a PhD in cardiovascular surgery. He is board-certified in cardiovascular surgery and has extensive experience with both open and endovascular procedures, including thoracic and abdominal endovascular aortic repair. His clinical and research interests focus on valve surgery, ViV transcatheter aortic valve implantation, structural heart disease, and complex aortic interventions. He is actively involved in clinical research, surgical education, and academic activities within cardiovascular and vascular surgery societies.

## Notes

**Consent:** Written informed consent for publication of this case report and accompanying images was obtained from the patient in accordance with the COPE guidelines.
